# New Appliances and Things Medical

**Published:** 1899-08-26

**Authors:** 


### NEW APPLIANCES AND THINGS MEDICAL.

[We shall be glad to receive, at our Office, 28 & 29, Southampton Street, Strand, London, W.C., from the manufacturers, specimens of all new preparations and appliances which may be brought out from time to time.]

### VARALETTES.

(ALFRED BISHOP, LIMITED, MILE END NEW TOWN, LONDON, E.C.)

IN a recent issue we drew attention to certain varalettes which had particular value as solvents of uric acid, and among them we mentioned those containing piperazine citrate and urotropine. We have now examined some additional varieties belonging to the mineral water series, namely, Carlsbad, Kissengin, and Vichy. The incorporation of the salts contained in these springs in such compact and handy little tabloids is a most convenient and economical method of bringing the advantages of the respective waters within the reach of the stay-at-home public. The varalettes are put up in bottles containing 50 in each, and the effervescent mixture which is produced by dissolving a few in a tumbler of water is decidedly more agreeable to the taste than taking an equal quantity of the natural water. Two varalettes of Carlsbad salts dissolved in half a tumbler of hot water at a temperature of 162 deg. Fahr. represents an equal quantity of the natural water. At Carlsbad two or three doses of this quantity are taken every morning, with walking exercise in between. If a similar treatment be submitted to at home the greatest benefit will be derived from those suffering from obesity, gout, gravel, or diabetes. With regard to the Vichy varalettes, if two of these be dissolved in half a tumbler of water of 106 deg. Fahr., practically an exact equivalent of the natural water is obtained. Being a mild alkaline water, large quantities can be taken with impunity, and with great benefit in conditions of congestion of the internal organs, and it is of special value when the liver or kidneys are so affected. The Kissengin varalettes should be dissolved in water at the low temperature of 51 deg. and in the same proportion as the two above-mentioned varieties. Owing to the energy of this enterprising firm almost any well-known mineral water can be prepared in this extremely cheap and convenient form. We therefore strongly recommend those who require a course of mineral waters and are unable to pay a visit to the natural course of the waters to make a conscientious and systematic trial of them by the exceeding convenient method invented by Alfred Bishop and Co.

